# P-1992. Significant Positive Correlation of PD-1 and PD-L1 Levels in Children with Post-acute Sequelae of SARS-CoV-2 Infection in Taiwan

**DOI:** 10.1093/ofid/ofae631.2150

**Published:** 2025-01-29

**Authors:** Yu-Lung Hsu, Pei-Chi Chen, Lawrence Shih-Hsin Wu, Kai-Sheng Hsieh, Huan-Cheng Lai, Hsiao-Chuan Lin, Kao-Pin Hwang, Jiu-Yao Wang

**Affiliations:** Division of Pediatric Infectious Diseases, China Medical University Children’s Hospital, China Medical University, Taichung, Taiwan, Taichung, Taichung, Taiwan (Republic of China); Center for Allergy, Immunology, and Microbiome (A.I.M.), China Medical University Hospital, China Medical University Children’s Hospital, China Medical University, Taichung, Taichung, Taiwan; Graduate Institute of Biomedical Sciences, China Medical University, Taichung, Taichung, Taiwan; Department of Medical Research, China Medical University Children’s Hospital, China Medical University, Taichung, Taichung, Taiwan; China Medical University Children’s Hospital, China Medical University, Taichung, Taichung, Taiwan; China Medical University Children’s Hospital, China Medical University, Taichung, Taichung, Taiwan; Division of Pediatric Infectious Diseases, China Medical University Children's Hospital, China Medical University, Taichung, Taiwan; School of Medicine, China Medical University, Taichung, Taiwan, Taichung City, Taichung, Taiwan; Center for Allergy, Immunology, and Microbiome (A.I.M.), China Medical University Hospital, China Medical University Children’s Hospital, China Medical University, Taichung, Taichung, Taiwan

## Abstract

**Background:**

Post-acute sequelae of SARS-CoV-2 infection (PASC) is a condition that impacts certain children following recovery from COVID-19. Programmed death-1 (PD-1) and its ligands, PD-L1 and PD-L2, may play a critical role in the immune response in PASC. However, the specific functions of these components in PASC among pediatric patients remain unclear.

**Figure:**
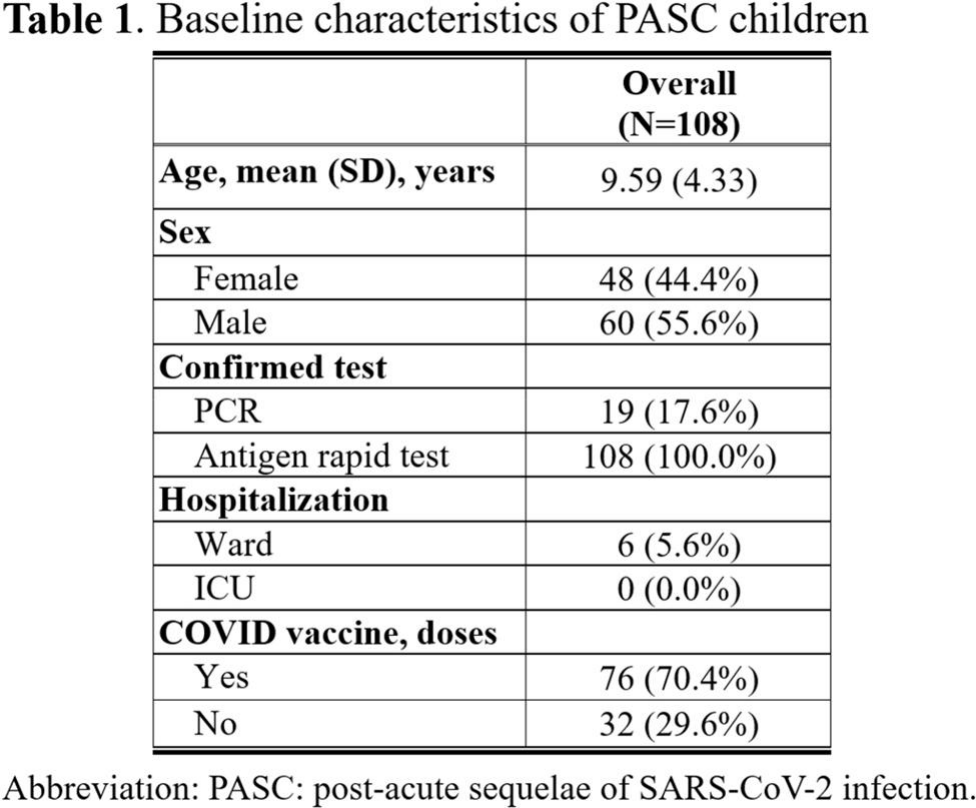

**Methods:**

The study participants were recruited from the DISCOVER (Diagnosis and Support for COVID Children to Enhance Recovery) study, conducted at the China Medical University Children's Hospital in central Taiwan during the Omicron outbreak. Children aged 3-18 years, previously diagnosed with SARS-CoV-2 infection via polymerase chain reaction (RT-PCR) or antigen rapid tests and identified specifically for post-acute sequelae of SARS-CoV-2 infection (PASC) in the outpatient department, were enrolled from October 1, 2022, to August 15, 2023. The investigation assessed the clinical characteristics of children with PASC, including measuring serum levels of PD-1, PD-L1, and PD-L2 to examine their interrelationships and the influence of demographic factors.

**Figure 1.**
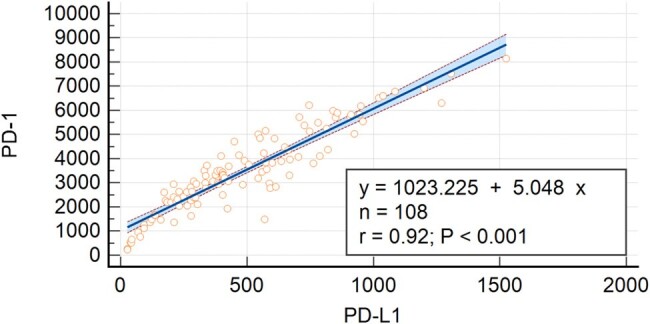
Correlation between PD-1 and PD-L1 levels in PASC children

**Results:**

Baseline characteristics of the study cohort (N=108) indicated an average age of 9.59 years, with 55.6% being male. All children had confirmed infections via antigen rapid tests, with 70.4% vaccinated against COVID-19. Only a small proportion (5.6%) were hospitalized, and none required ICU care. Our analysis identified a significant positive correlation between PD-1 and PD-L1 concentrations (r=0.92, P< 0.001) in children with PASC. In contrast, there was no significant relationship between PD-1 and PD-L2 (r=0.14, P=0.138). There were no significant differences in PD-1 concentrations across different age groups, genders, vaccination statuses, or various PASC symptoms.

**Figure 2.**
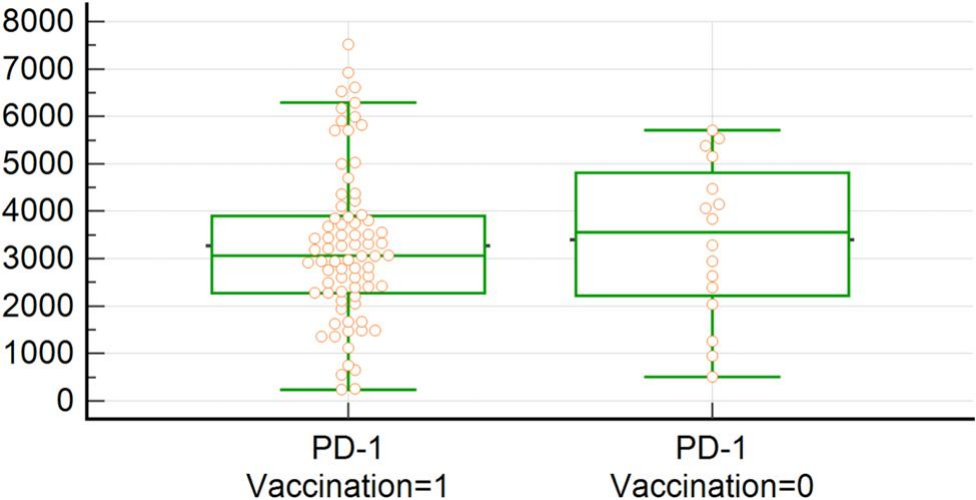
PD-1 level in PASC children with or without COVID-19 vaccination

**Conclusion:**

The significant correlation between PD-1 and PD-L1 highlights a potentially enhanced immunosuppressive state in children after COVID-19, which might predispose them to PASC. Our findings suggest the need for further investigation into the PD-1/PD-L1 axis to fully understand its implications in this vulnerable population.

**Figure:**
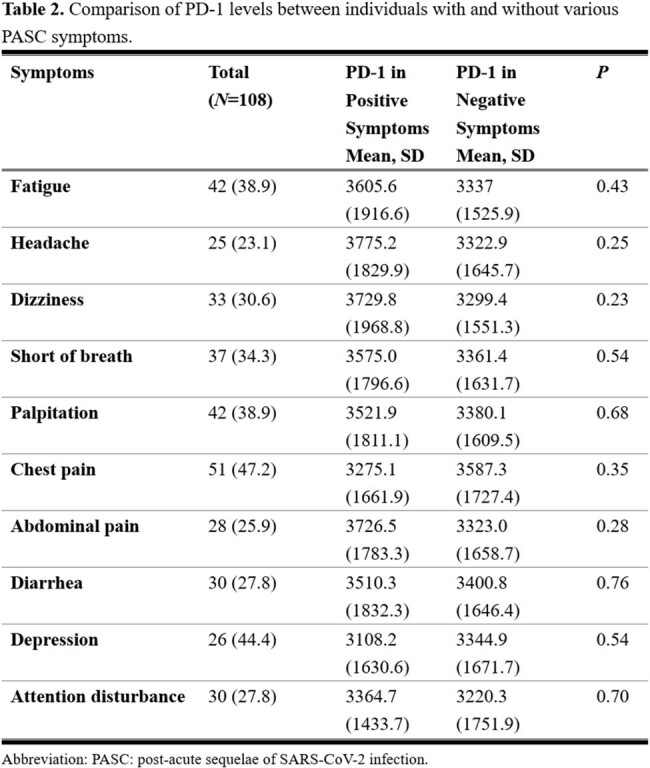

**Disclosures:**

All Authors: No reported disclosures

